# Continuous evolution of influenza A viruses of swine from 2013 to 2015 in Guangdong, China

**DOI:** 10.1371/journal.pone.0217607

**Published:** 2019-07-19

**Authors:** Zhenpeng Cao, Weijie Zeng, Xiangqi Hao, Junming Huang, Mengkai Cai, Pei Zhou, Guihong Zhang

**Affiliations:** 1 College of Veterinary Medicine, South China Agricultural University, Guangzhou, China; 2 National Engineering Research Center for Breeding Swine Industry, Guangzhou, China; 3 Key Laboratory of Zoonosis Prevention and Control of Guangdong Province, Guangzhou, China; University of Illinois College of Medicine, UNITED STATES

## Abstract

Southern China is considered an important source of influenza virus pandemics because of the large, diverse viral reservoirs in poultry and swine. To examine the trend in influenza A virus of swine (IAV-S), an active surveillance program has been conducted from 2013 to 2015 in Guangdong, China. The phylogenetic analyses showed that the external genes of the isolates were assigned to the Eurasian avian-like swine (EA) H1N1 and/or human-like H3N2 lineages with multiple substitutions, indicating a notable genetic shift. Moreover, the internal genes derived from different origins (PB2, PB1, PA, NP: pdm/09 (pandemic influenza virus 2009)-origin, M: pdm/09- or EA-origin, NS: North American Triple Reassortant (TR)-origin have become the dominant backbone of IAV-S in southern China. According to the origins of the eight gene segments, the isolates can be categorized into five genotypes. The results of mice experiment showed that the YJ4 (genotype 1) and DG2 (genotype 4) are the most pathogenic to mice, and the viruses are observed in kidneys and brains, indicating the systemic infection. The alterations of the IAV-S gene composition supported the continued implementation of the intensive surveillance of IAV-S and the greater attention focused on potential shifts toward transmission to humans.

## Introduction

Influenza A virus (IAV) belongs to the *Orthomyxoviridae* family and contains a genome composed of eight single-stranded RNA genomes. According to the antigenic properties of hemagglutinin (HA) and neuraminidase (NA), IAV can be subtyped into 16 HA and 9 NA types in aquatic birds, and 2 HA and 2 NA subtypes have been detected in bats [1, 2]. Pigs play a pivotal role in the circulation and evolution of IAVs and are regarded as “mixing vessels” for the generation of novel reassortant viruses [3]. Three main subtypes (H1H1, H1N2 and H3N2) have been detected in pigs [4]. The simple infection of pigs with influenza A virus presents mild clinical signs in the field and under experimental conditions [5]. However, influenza A virus of swine (IAV-S) can impair the host immune system in a variety of ways, subsequently suppressing the immune response to other pathogens [6].

Multiple lineages of IAV-S have emerged and became established in pigs. Classical swine influenza virus (CS) and Eurasian avian-like swine influenza virus (EA) have been isolated in pigs in the 1930s and 1970s, respectively [7, 8]. Additionally, cases of pig infection by H1N1 and H3N2 human seasonal (HS) influenza viruses and avian influenza viruses (AIVs) have been sporadically reported worldwide [9–11], increasing the IAV-S gene pool and giving rise to numerous reassortant viruses such as the North American triple reassortant (TR) influenza virus [12, 13]. In April 2009, a reassortant variant, pandemic influenza virus 2009 (pdm/09), sustained human-to-human transmission and caused the first pandemic in the 21^st^ century. This variant possessed TR-origin PB2, PB1, PA, NP, HA (H1), and NS genes and EA-origin NA (N1) and M genes [14].

After the pandemic, the 2009 pandemic virus was repeatedly transmitted to pigs in many countries [15–17] and reassorted with endemic viruses [18, 19], which increased the genetic diversity of IAV-S circulating in pig herds. Phylogenetic and genetic analyses revealed that pdm/09-origin internal genes became established and evolved variants [20]. To research the trend in IAV-S in pig herds, an active surveillance program has been conducted in pigs from 2013 to 2015 in Guangdong, China. The origin, gene diversity and genetic markers of the isolates in this program were estimated through phylogenetic and molecular analysis. Mouse is one of the most mature mammal models for influenza virus infection, which has been used for evaluating the virulence of avian- and mammal-origin influenza viruses [21]. The pathogenicity of the strains to mice has been assessed in this study.

## Materials and methods

### Sample collection and virus isolation

An active surveillance program of IAV-S in pig herds has been performed from 2013 to 2015. The nasal swabs utilized in this program were collected from commercial farms and abattoirs in Guangzhou, Foshan, Dongguan, Yangjiang, and Jiangmen, among other locations in China. The samples were transported to the laboratory at 4°C with ice bags and preserved at −80°C. The swabs were inoculated into 9–11 days old specific-pathogen-free (SPF) embryonated eggs and/or Madin-Darby Canine Kidney(MDCK) cell cultures in Eagle's minimum essential medium with trypsin (2 μg/ml). The embryonated eggs incubated at 37°C for 72h and the cell culture tubes were incubated for up to 7 days. Virus isolates were passaged and identified using hemagglutination test with chicken red blood cells. The positive allantoic fluid and/or supernatant were collected were used to extract the viral RNA for whole genome sequencing.

### RT-PCR and sequencing

Reverse transcription-polymerase chain reaction (RT-PCR) was performed to amplify the viral RNA for sequencing and phylogenetic analysis. Viral RNA was extracted using TRIzol^TM^ Reagent (Thermo Fisher Scientific, USA) and amplified in one reaction with reverse transcriptase M-MLV (RNase H-), recombinant RNase inhibitor (TaKaRa, Japan) and the oligonucleotide universal primer 5’-AGCAAAAGCAGG-3’. The genome was amplified by PCR using the Platinum^TM^ Pfx DNA polymerase kit (Thermo Fisher Scientific, USA) with a series of primers[22]. The products were then purified using an agarose gel DNA purification kit (TaKaRa, Japan). To sequence the genes, the products were cloned into the pJET 1.2 blunt-end cloning vector (Thermo Fisher Scientific, USA) and transformed into competent DH5α (Tiangen Biotech Beijing Co., Ltd.). At least three clones per gene were sequenced by Sanger sequencing (Thermo Fisher Scientific, USA).

#### Molecular analysis

The sequences were compiled and edited using SeqMan and Editseq (DNASTAR, Lasergene. v7.1). BLAST analysis was carried out using NCBI. The sequences of the isolates were uploaded to the GISAID Epiflu database. The consensus sequences referenced in this study were downloaded from GenBank and the GISAID Epiflu database, purged for redundant sequences (>99.9% similarity), and aligned using MAFFT [23] and further edited using MEGA version 5 [24]. The number of amino acid residues of HA (H1 and H3 numbering after removal of the signal peptide) and other proteins (open reading frame, ORF) was based on the downloaded sequences. Potential asparagine-linked glycosylation sites (PNGs) in the HA proteins were predicted by examining the N–X–S/T motifs using the NetNGlyc 1.0 Server, and X could be any amino acid except proline (http://www.cbs.dtu.dk/services/NetNGlyc/).

#### Phylogenetic analysis

A total of 872 sequences, 70–100 sequences per gene segment, were processed for phylogenetic analysis. These sequences included several endemic strains, mainly in mainland China and Hong Kong. The analysis were based on the following sequences: H1 HA, nt 33–1733; H3 HA, nt 30–1730; N1 NA, nt 21–1430; N2 NA, nt 20–1429; PB2, nt 28–2307; PB1, nt 25–2298; PA, nt 25–2175; NP, nt 46–1542; M, nt 26–1007; NS, nt 27–864.

The best-fit nucleotide substitution model was predicted using ModelTest [25]. The time of the most recent common ancestor (tMRCA) was determined using the Bayesian Markov Chain Monte Carlo (MCMC) method in BEAST v1.8.3 [26, 27], under the HKY substitution model with gamma distributed rates amongst sites (HKY+γ), using a Bayesian skyline coalescent tree [28] prior to selecting a relaxed uncorrelated lognormal model [29]. MCMC chains were run for 5~10×10^7^ generations for each dataset. The tMRCA was estimated using the collection date adjusted to year format in this study. Tracer v1.6 was used for evaluating the MCMC output results by caculating the the ESS (effective sample size) [30]. The trees were generated using TreeAnnotator v1.8.3 (http://beast.bio.ed.ac.uk/TreeAnnotator) with 10% burn-in and 95% highest probability density (HPD) values. The trees were visualized and redacted using FigTree v1.4 (http://beast.bio.ed.ac.uk/figtree).

### Mice experiment

6-week-old female BALB/c mice (Guangdong Medical Laboratory Animal Center, Guangdong, China) were used in this study. Thirteen mice per group were anaesthetized with dry CO_2_ and inoculated intranasally with 10^6.0^ EID_50_ viruses in a volume of 50μL. Three mice were euthanized on day 3 post-inoculation with dry CO_2_, and the virus titrations of the lungs, kidneys, brains, and turbinates were tested in chicken embryos eggs. The samples were homogenized in 1 mL per 1g tissue of PBS supplemented with penicillin (1,000 U/mL) and streptomycin (1,000 U/mL) and were centrifuged at 4,000 ×g to isolate supernatant fluids. The supernatants were serially diluted and inoculated into the allantoic cavity of 9–10 days old chicken embryonated eggs (100 μL per egg). The eggs were incubated at 37°C for 48 h. The virus titers were detected by the hemagglutina tion test and calculated using the method of Reed and Muench method. The weight loss and survival of the remaining ten mice were recorded daily for a total of 14 days.

The animal experiments were carried out in ABSL-3 facilities in compliance with the biosafety committee of South China Agriculture University (SCAU) protocols. All animal experiments were approved by the Institutional Animal Care and Use Committee at SCAU and carried out in accordance with the approved guidelines. Animals determined to have lost > 25% of body weight or to be moribund (as indicated by increased respiratory rate and inability to ambulate) were euthanized.

## Results

### Nasal swab collection and virus isolation

From June 2013 to December 2015, a total of 4,056 swine nasal swabs were collected randomly from 35 commercial farms and 5 abattoirs in Guangdong, China. The swabs were collected from Guangzhou (n = 1276), Foshan (n = 1180), Dongguan (n = 225), Yangjiang (n = 458), Jiangmen (n = 278) and Shenzhen (n = 639). Fourteen viruses were isolated using chicken embryos and/or MDCK cells and were identified using the hemagglutination test and were confirmed by RT-PCR and genomic sequencing. The sequences of the isolates have been uploaded to the GISAID Epiflu database, and the available IDs were listed in Table 1. According to the results of BLAST, the isolates can be categorized into three subtypes, including eight H1N1 stains, five H1N2 strains and one H3N2 strain.

**Table 1:** Novel reassortant viruses isolated in this study.

Isolate	Abbreviation^a^	Subtype	District	Source^b^	Collection date	GISAID isolate_ID^c^
A/Swine/Guangdong/DL2/2013	DL2	H1N1	Foshan	Farm	Jul. 17th, 2013	EPI_ISL_249793
A/Swine/Guangdong/CH7/2014	CH7	H1N2	Guangzhou	Farm	Nov. 12th, 2014	EPI_ISL_249794
A/Swine/Guangdong/CH8/2014	CH8	H1N1	Guangzhou	Farm	Nov. 12th, 2014	EPI_ISL_249795
A/Swine/Guangdong/EP/2014	EP	H1N2	Jiangmen	Farm	Dec. 3rd, 2014	EPI_ISL_249796
A/Swine/Guangdong/YJ4/2014	YJ4	H1N1	Yangjiang	Farm	Dec. 26th, 2014	EPI_ISL_249797
A/Swine/Guangdong/YJ10/2014	YJ10	H1N1	Yangjiang	Farm	Dec. 26th, 2014	EPI_ISL_249798
A/Swine/Guangdong/YJ23/2014	YJ23	H1N1	Yangjiang	Farm	Dec. 26th, 2014	EPI_ISL_249799
A/Swine/Guangdong/YJ28/2014	YJ28	H1N2	Yangjiang	Farm	Dec. 26th, 2014	EPI_ISL_249800
A/Swine/Guangdong/DG1/2015	DG1	H1N2	Dongguan	Farm	Oct. 15th, 2015	EPI_ISL_249801
A/Swine/Guangdong/DG2/2015	DG2	H1N2	Dongguan	Farm	Oct. 15th, 2015	EPI_ISL_249810
A/Swine/Guangdong/BRT15/2015	BRT15	H1N1	Foshan	Farm	Oct. 25th, 2015	EPI_ISL_249812
A/Swine/Guangdong/FNPA/2015	FNPA	H1N1	Foshan	Abattoir	Dec. 18th, 2015	EPI_ISL_249844
A/Swine/Guangdong/FS4/2015	FS4	H3N2	Foshan	Farm	Dec. 18th, 2015	EPI_ISL_249845
A/Swine/Guangdong/FSC17/2015	FSC17	H1N1	Foshan	Abattoir	Dec. 18th, 2015	EPI_ISL_249852

^a^ District abbreviation: DL, Dali in Foshan; CH, Conghua in Guangzhou; EP, Enping in Jiangmen; YJ, Yangjiang; DG, Dongguan; NP, Nanping in Foshan.

^b^ The soruce of the nasal swabs collected was commercial farm or abattoir.

^c^ Sequence information was generated and deposited in GISAID.

### Analysis of H1 HA

**Fig 1:**
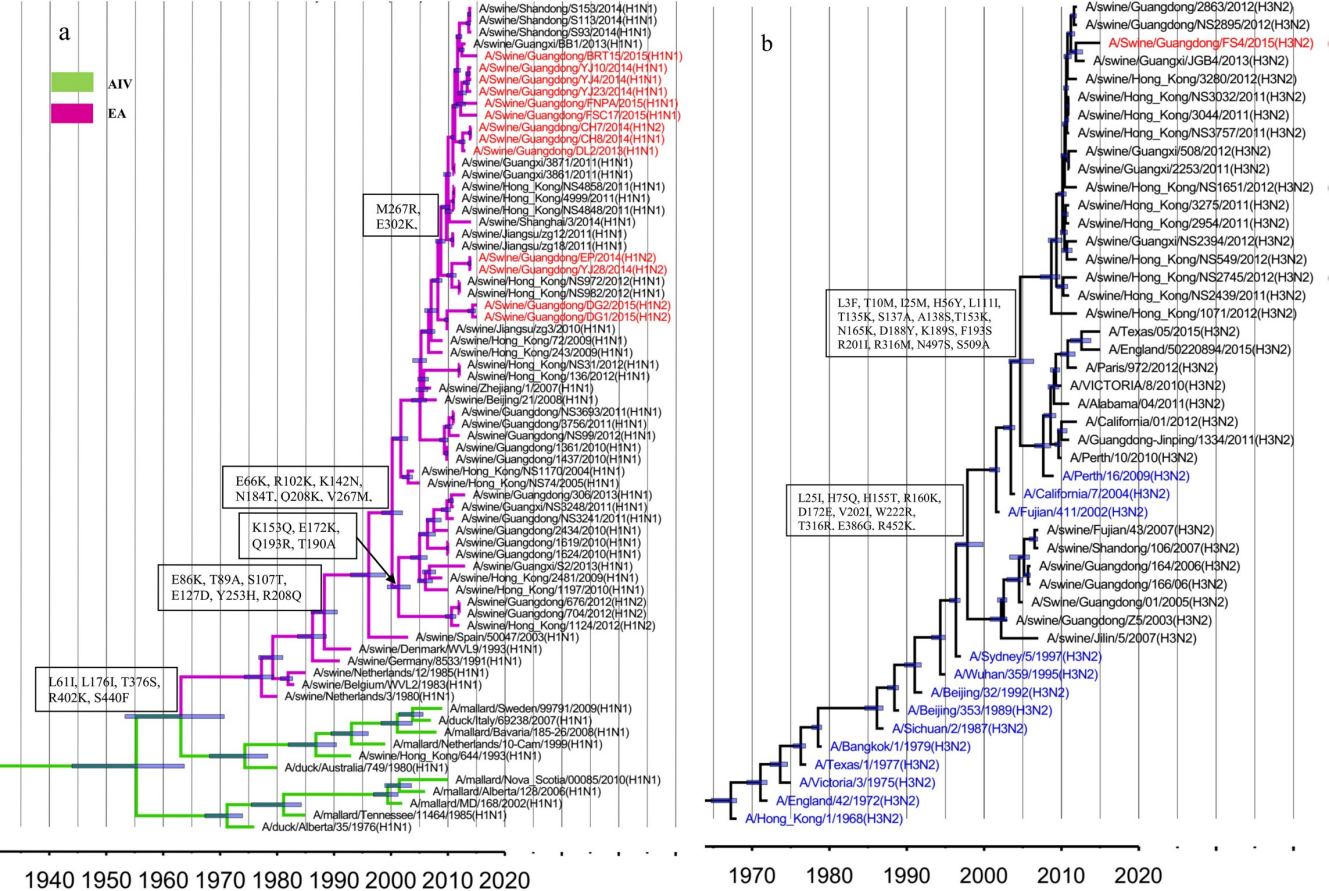
Genetic relationship of the H1- and H3- HA genes. The novel viruses investigated in this study are indicated by red labels, 95% highest probability density (HPD) values of the tMRCA are denoted by blue bars, and the main genetic markers between the sublineages are represented in the boxes. (a) H1 HA. The AIV and EA lineages are represented by green and purple branches, respectively. (b) H3 HA. Thirteen major genetic clusters (blue) are indicated in the H3 HA phylogenetic tree.

Among the 14 isolates identified in this survey, 13 HA genes belonged to the H1 subtype (Table 1), shared 94.7–99.9% nucleotide sequence identity and were assigned to the EA lineage (Fig 1A, red). The EA-origin viruses isolated from pigs in China after 2005 can been divided into five sublineages: Sw/HK/1124 (A/swine/Hong Kong/1124/2012)-like, Sw/HK/2481(A/swine/Hong Kong/2481/2009)-like, Sw/HK/1619(A/swine/Hong Kong/1619/2010)-like, Sw/HK/1361(A/swine/Hong Kong/1361/2010)-like and Sw/HK/72(A/swine/Hong Kong/72/2009)-like (Fig 1A, purple). Among these sublineages, multiple substitutions were observed in the antigenic sites, including E155G (H1 numbering) in Sa, N184T, A190T and R193Q in Sb, and K142N in Ca. Furthermore, many substitutions were identified in the HA1 protein, such as R102K, E172K, Q208K (Fig 1A), and no studies have shown whether these substitutions affect the virus characteristics.

**Fig 2:**
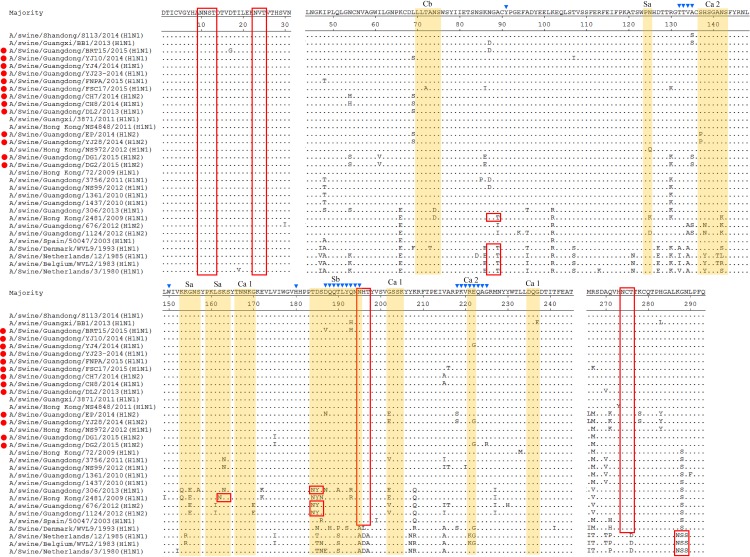
Amino acid alignment of H1 HA1 proteins without the signal peptide. Dots indicate amino acids that are identical to those in the consensus sequence. The predicted antigenic sites Sa, Sb, Ca and Cb were depicted with colored shading and listed on the top. PNGs are indicated by red boxes. The isolates analyzed in this study were labelled by red dots(●). Residues marked with blue inverted triangles (▼) are receptor-binding sites (RBS).

The H1HA genes in this study were all assigned to the Sw/HK/72-like sublineage and showed several unique substitutions in the antigenic sites (Fig 2), such as G202E in EP and YJ28, N194H in BRT15, and T72A in FSC17. At the receptor binding pocket, amino acid residues 225G were conserved in all H1 subtype isolates in this program and predicted to have no affinity for mammalian cell-surface receptors [31]. The N-linked glycosylation is essential for protein folding and maturation. Changes in the number or location of NLG sites in the HA protein can affect the biological activity of IAV. Five potential Potential asparagine-linked glycosylation sites N-linked glycosylation sites(PNGs) were conserved in all Chinese EA-origin viruses isolated since 2005, including 10NNS12, 11NST13, 23NVT25, 195NHT197 and 274NCT276. However, Sw/HK/2481-like viruses included 87NGT89 and 162NKS164 due to the A89T and S162N substitutions, and the Sw/HK/1124-like and Sw/HK/1619-like viruses included 184NYS186 because of the T184N substitution. In this study, EP and YJ28 were distinct from the other isolates and showed S137P in antigenic site Ca and P218S, M266L, K278S substitutions. Moreover, many novel substitutions were found in the novel isolates, such as N54S, I61V, K86E, A135S, V175I in DG1 and DG2, D187V, Q193H in BRT15, and E222G in YJ4, YJ28 and DG2 (Table 2).

**Table 2:** The substitutions of the novel isolates.

Segment		Substitution ^a^
HA	H1	N54S, I61V,T72A, K86E, S137P, A135S, V175I, T184N, D187V, Q193H, N194H, G202E, P218S, M266L, K278S,
	H3	S45N, T135K, N165K, G218V, G275D
NA	N1	I17T, N42D, S46P, S70N, S95N, R130K, K217R, I255V, E287K, I289T, N307D, D316G, T332I, T332V, P340S, R382G, R430Q
	N2	R39Q, P46S, M51T, T71N, K80Q, K136R, V165I, K172R, R224T, E258K, Q273K, I312T, H336Y, N385K, D399E, K415N, T434P,
PB2		R8K, K126R, A221V, E249D, R251K, I299V, K389R, K412R, E681D, D701N, S714G,
PB1		P64S, N158S, N175D, G216D, I336V, E371D, P454S, Y483N,
PA		A70V, K256Q, D272E, R356K, N364S, H437I, T639A
NP		A27S, P95S, C164Y, V186I, M316I, K351R, T396N
M		S30N, R105G, T154N in pdm/09-like M1 proteins and I28T, V27A, P35A in pdm/09-like M2 proteins; N36S in EA- like M1 proteins and N13S, L55F in EA-like M2 proteins
NS		L15I, T18V, N48S, V60I, Q109H, H169I, P216S in NS1 proteins and M31I and V32I, T48S, E67K, K88R in NEP

^a^, the substitutions were found in at least one isolate

### Analysis of the FS4 HA

The remaining FS4 HA gene belonged to the H3 subtype. To assess its origin, 13 major human-origin H3 HA genetic clusters that had been circulating since 1968 were estimated [32] and represented in the H3 HA phylogenetic tree (Fig 1B, blue labels). The results showed that the H3 HA genes isolated after 2009 could be divided into two distinct sublineages based on the referenced sequences, Hu/Perth/16(A/Perth/16/2009)-like and Sw/HK/1071(A/swine/Hong Kong/1071/2012)-like. Several substitutions at antigenic sites were observed between Hu/Perth/16-like and Sw/HK/1071-like, including T135K (H3 numbering), S137A, I140K and R143G in site A, D188Y, K189S, F193S in site B, E50G in Site C and R201I in site D (Fig 1B).

**Fig 3:**
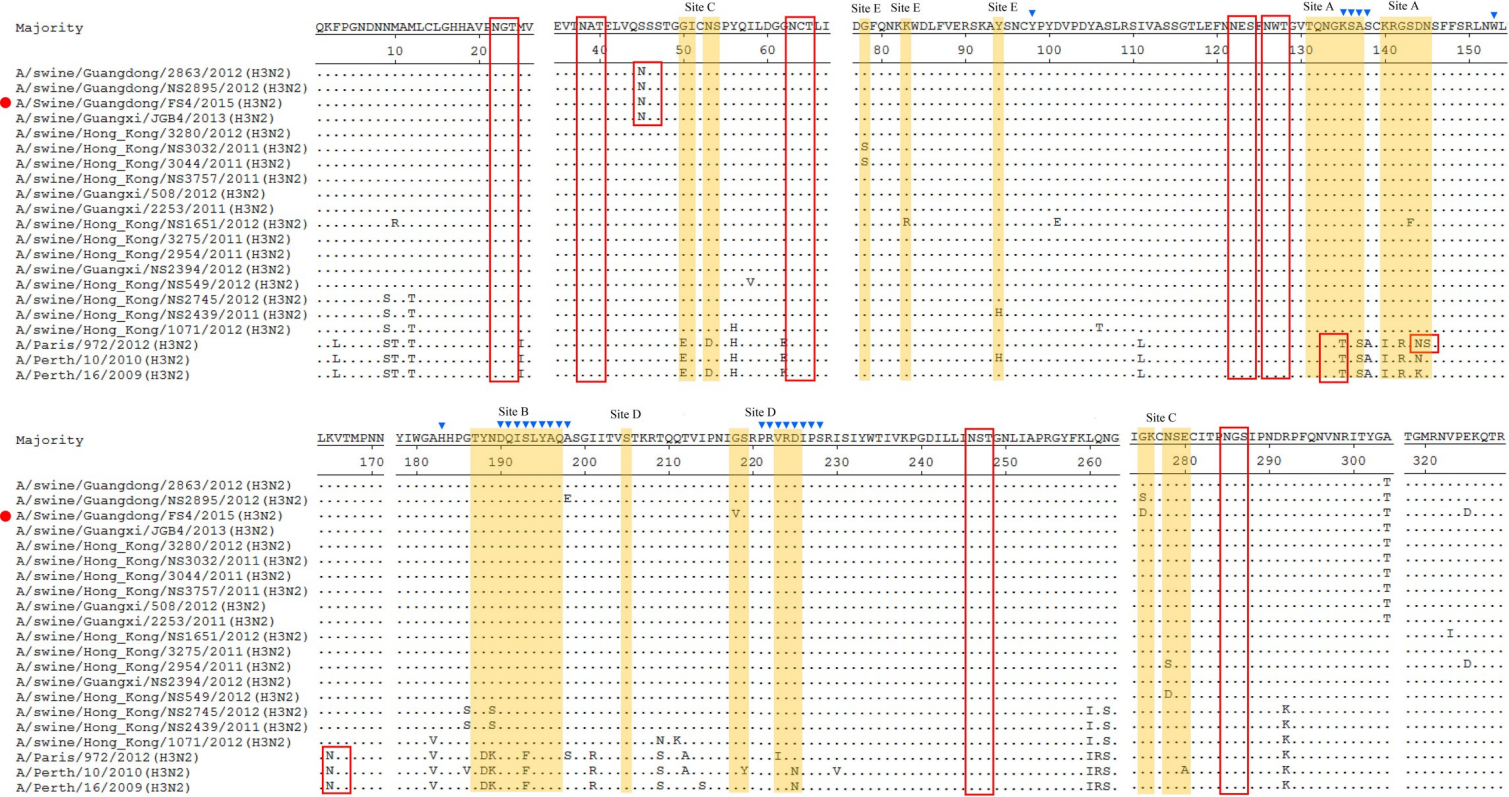
Amino acid alignment of FS4 HA1 without the signal peptide. The predicted antigenic sites A, B, C, D and E defined previously are depicted with colored shading and listed on the top. The other markers are indicated as in Fig 2.

As shown in the phylogenetic tree, the FS4 HA gene was assigned to the Sw/HK/1071-like sublineage (Fig 1B, red), and substitutions such as G275D in site C and G218V in site D were found in the HA1 protein (Fig 3). With respect to receptor binding affinity, 226I and 228S in H3 HA at the receptor binding pocket were fairly conserved in FS4 and are usually detected in human viruses. Seven PNGs (22NGT24, 38NAT40, 63NCT65, 122NES124, 126NWT128, 246NST248 and 285NGS287) were present in the Hu/Perth/16-like and Sw/HK/1071-like viruses. The T135K and N165K substitutions, which eliminated two potential glycosylation sites at position 133NGT135 and 165NVT167, were observed in Sw/HK/1071-like viruses. In this study, a new PNG was observed at position 45 in FS4 for the S45N substitution (Fig 3).

### Phylogenetic and molecular analysis of NA genes

**Fig 4:**
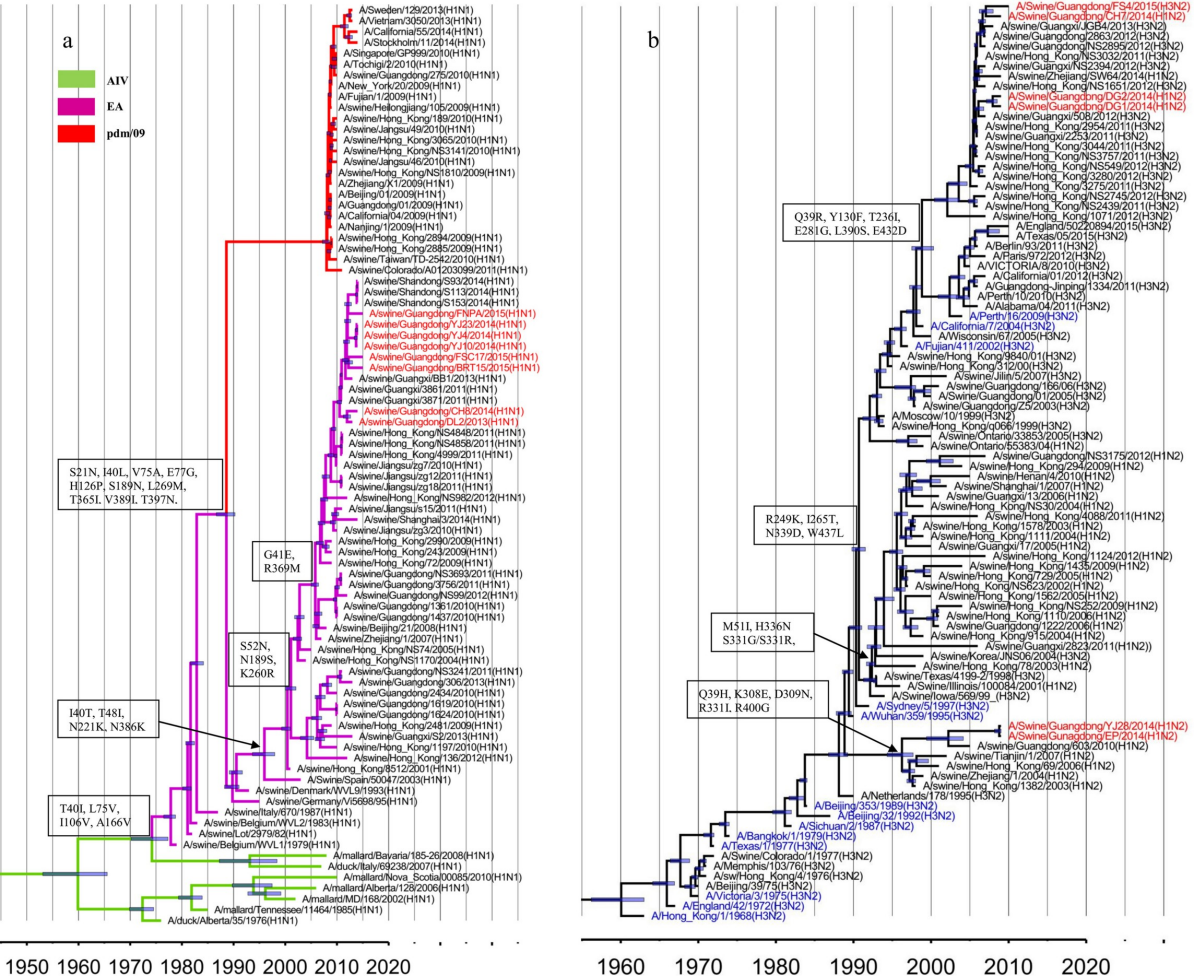
Phylogenetic analysis of NA genes. **(a) N1 NA.** The branches, labels, genetic markers and 95% HPD of the tMRCA are represented as in Fig 1. The red branches indicate the pdm/09 lineages. **(b) N2 NA.** The labels, genetic markers and 95% HPD values of the tMRCA are represented as in Fig 1.

Eight NA genes shared 96.7–99.9% sequence identity and belonged to the EA lineage (Fig 4A, red). Similar to the EA-origin HA genes, the EA-origin NA genes differentiated from the AIV lineage and are prevalent in pig herds (Fig 4A). The EA-origin NA genes isolated in China since 2000 exhibited I40T (N1 Numbering), T48I, N221K, N386K substitutions and differentiated into two main sublineages, termed Sw/HK/2481-like and Sw/ZJ/1 (A/swine/Zhejiang/1/2007)-like. As shown in the phylogenetic tree, all N1 NA genes in this study were assigned to the Sw/ZJ/1-like sublineage. The DL2 and CH8 NA genes were distinct from the rest of the isolates and showed several unique substitutions, including S46P, I255V, E287K, I289T and P340S. Furthermore, multiple substitutions were observed in the N1 NA genes of the isolates, such as the N42D and R130K substitutions in CH8, S95N, and K217R in YJ10, I17T, S70N, T332I and D316G in BRT15, and N307D, T332V, R382G and R430Q in FSC17 (Table 2).

According to the referenced sequences, the N2 NA genes were divided into four main sublineages, which were designated Sw/HK/1382 (A/swine/Hong Kong/1382/2003)-like, Sw/HK/78 (A/swine/Hong Kong/78/2003)-like, Hu/Perth/16-like, and Sw/HK/1071-like (Fig 4B). The N2 NA genes in this study shared 89.7–99.2% nucleotide sequence identity and were assigned to two distinct sublineages. The EP and YJ28 NA genes were assigned to the Sw/HK/1382-like lineage and showed P46S, T71N, V165I, K172R, E258K, D399E and K415N substitutions (Table 2). The CH7, FS4, DG1 and DG2 NA genes were assigned to the Sw/HK/1071-like lineage (Fig 4B). Several unique substitutions, such as M51T, K136R, H336Y, and T434P were observed in DG1 and DG2. And the R39Q, K80Q, R224T, Q273K, I312T and N358K substitutions were found in the FS4 NA gene(Table 2).

### Phylogenetic analysis of the internal genes

**Fig 5:**
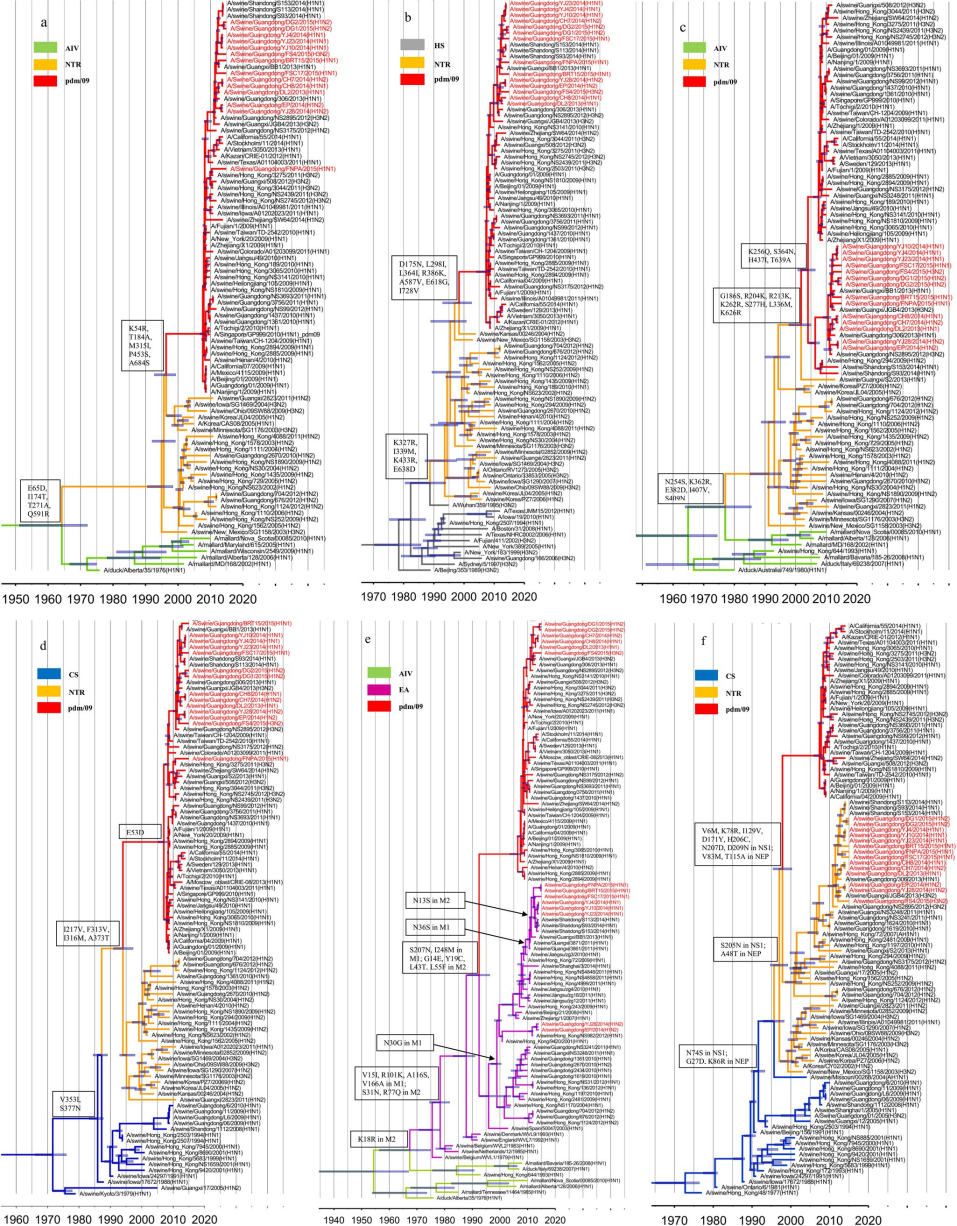
**Genetic relationships of the PB2 (a), PB1 (b), PA (c), NP (d), M(e) and NS(f) genes.** The genetic markers, labels and 95% HPD of the tMRCA are represented as in Fig 1. The representative strains of the EA lineage are depicted in orange. The red, green, purple, blue, gray and orange branches represent the pdm/09, AIV, EA, CS, HS and TR lineages, respectively.

The PB2, PB1, PA and NP gene segments of the novel isolates shared 96.5–99.9%, 97.7–100.0%, 97.7–99.9% and 96.4–100.0% nucleotide sequence identity, respectively. All of these genes were assigned to the pdm/09 lineage (Fig 5A, 5B, 5C and 5D, red), which is related to the TR lineage.

The V255I, R389K, and V731I substitutions were identified in the pdm/09-origin PB2 genes isolated since 2013. However, the predominant sites in the main functional domains were relatively conserved after 2009. For example, 627E was present in the pdm/09-origin PB2 gene segment, which is regarded as a determinant of host range and is present in the majority of avian viruses [33]. All isolates except FNPA were related to A/Swine/Guangxi/BB1/2013, and multiple substitutions were found in the PB2 genes of the novel isolates, including R8K and S714G in DL2, CH7 and CH8 and A221V, E249D and R251K in YJ4, YJ10, and YJ23 (Table 2). Interestingly, the PB2 gene of FNPA was distinct from the other isolates and contained the K126R, I299V, K389R, K412R, E681D and D701N substitutions (Table 2). The pdm/09-origin PB1 genes isolated since 2011 contained several novel substitutions, including I12V, V517I, D377E, I435V and A652V. Three genetic markers, P64S, I336V and P454S, were present in the PB1 genes of YJ4, YJ10 and YJ23 but not in viruses isolated in 2015 (Table 2). Furthermore, substitutions such as N175D and G216D in DG1 and DG2, Y483N in DG2, and N158S and E371D in FNPA were found in these novel isolates (Table 2). The pdm/09-origin PB1 genes, including those of the novel isolates, had a stop codon (TAA) at nucleotide position 128, indicating a truncation of PB1-F2 after 11 amino acids.

The pdm/09-origin PA and NP genes were divided into two distinct sublineages and likely emerged in 2004 and 2007, respectively, as indicated by the time of the most recent common ancestor (tMRCA). Two sublineages were denoted the Sw/GD/306 (A/swine/Guangdong/306/2013)-like and Hu/GD/01(A/ Guangdong/01/2009)-like lineages, respectively. In this study, all PA and NP genes were assigned to the Sw/GD/306-like sublineage. The PA genes of the novel isolates contained the K256Q, H437I, T639A substitutions (Table 2). Furthermore, multiple unique substitutions were found in the isolates, such as R356K in DL2, CH7 and CH8, D272E and N364S in EP and YJ28, and A70V in YJ4, YJ10 and YJ28. Regarding NP genes, V186I substitutions were observed in the novel isolates except DG2, and P95S and M316I substitutions were detected in YJ4, YJ10, YJ23, BRT15 and FSC17. FNPA, which was distinct from the rest of the isolates, contained A27S, C164Y, K351R and T396N substitutions (Table 2).

The pdm/09-origin M genes approximately differentiated from the EA lineage 22 years prior (Fig 5E). The M genes of DL2, CH7, CH8, DG1, DG2 and FS4 belonged to the pdm/09 lineage, and the remaining isolates were assigned to the EA lineage (Fig 5E). Compared to the EA-origin M genes, the pdm/09-origin M contained multiple substitutions, including S207N and I248M in the M1 protein and G14E, Y19C, L43T, and L55F in the M2 protein (Table 2). The pdm/09-origin M genes in this study showed many novel substitutions, including S30N, R105G, T154N in the M1 protein and P35A and I28T in the M2 protein of FS4 and V27A in the M2 protein of DG1 and DG2 (Table 2). The EA-origin M genes in this study belonged to two distinct sublineages, which likely differentiated in 2000 as indicated by the tMRCA. The M genes of YJ28 and EP were assigned to the Sw/HK/9420 (A/swine/Hong Kong/9420/2001)-like sublineage and showed L55F in the M2 protein. The M genes of YJ4, YJ10, YJ23, BRT15, FNPA and FSC17 belonging to the Sw/ZJ/1-like sublineage, possessed an N36S substitution in M1 and an N13S substitution in M2 (Table 2).

The phylogeny of the NS genes differed from that of the rest of the internal genes. In this study, the NS genes shared 96.9–100.0% nucleotide sequence identity, and all belonged to the TR lineage (Fig 5F, red). As shown in the phylogenetic tree, the TR-origin NS genes were divided into two distinct sublineages, Hu/Korea/CY02 (A/Korea/CY02/2002)-like and Sw/HK/1562 (A/swine/Hong-Kong/1562/2005)-like, which were established in Korea and southern China, respectively. In this study, all NS genes were assigned to the Sw/HK/1562-like sublineage and displayed T18V, N48S, Q109H and P216S in NS1 and M31I and T48S in NEP (Table 2). Furthermore, several unique substitutions were observed in the NS genes of the isolates, such as L15I, V60I, H169I in NS1 and V32I, E67K, K88R in NEP of the novel isolates (Table 2).

### Drug resistance analysis

Oseltamivir is an important antiviral drug used in the treatment of infectious influenza viruses [34]. E119G/V, D199G, I223K/R/V, S247N, H275Y and N295S (N1 numbering) in N1NA, E119D/V, Q136K, I222L, R292K, N294S, and deletion of 245–248 (N2 numbering) in N2 NA have been detected in viruses associated with oseltamivir resistance (data reported in WHO). None of the above substitutions were detected in the N1 and N2 proteins of the isolates in this study, indicating their susceptibility to oseltamivir. Adamantanes, a group of antiviral drugs that inhibit the function of the viral M2 proton channel, are used for the treatment of human influenza. In our study, K, A and G were fairly maintained at positions 26, 30 and 34, respectively. However, N was found at position 31 of M2 protein in the isolates, and the V27A substitution was observed in DG1 and DG2; these substitutions confer resistance to amantadine [35].

### Classification of genotypes of the novel viruses

To describe the genetic diversity of the novel isolates, genotypes were defined on the basis of the original lineage of the eight gene segments. Phylogenetic analysis revealed that the genomes of these novel isolates were reassortant from three or four lineage origins, and divided into five distinct genotypes. All the isolates possessed the pdm/09-origin PB2, PB1, PA and NP gene segments, and TR-origin NS gene segment. Eight out of 14 isolates in this study possess the H1N1 EA-origin surface genes, and pdm/09-origin M genes (n 2) or the EA-origin M genes (n 6). Five H1N2 viruses isolated in this study possess the EA-origin HA (H1) gene and N2 human-origin NA gene (N2), with the pdm/09-origin M genes (n 3) or the EA-origin M genes (n 2). Only one H3N2 subtype influenza virus was isolated in this surveillance, possessing H3N2 human-originsurface gene segments, pdm/09-origin PB2, PB1, PA, NP and M gene segments, and TR-origin NS gene (Table 3).

**Table 3:** Identification of genotypes for novel isolates.

Genotype^a^	Isolates	HA	NA	PB2	PB1	PA	NP	M	NS
1	YJ4、YJ10、YJ23、BRT15、FNPA、FSC17	EA	EA	P	P	P	P	EA	TR
2	DL2、CH8	EA	EA	P	P	P	P	P	TR
3	EP、YJ28	EA	Sw-N2	P	P	P	P	EA	TR
4	CH7、DG1、DG2	EA	Sw-N2	P	P	P	P	P	TR
5	FS4	Sw-H3	Sw-N2	P	P	P	P	P	TR

^a^,Genotypes of the viruses are showed in the right with eight blocks representing each gene segment (from left to right, HA, NA, PB2, PB1, PA, NP, M, and NS). The strains are showed on the left.

### Pathogenicity in mice

**Fig 6:**
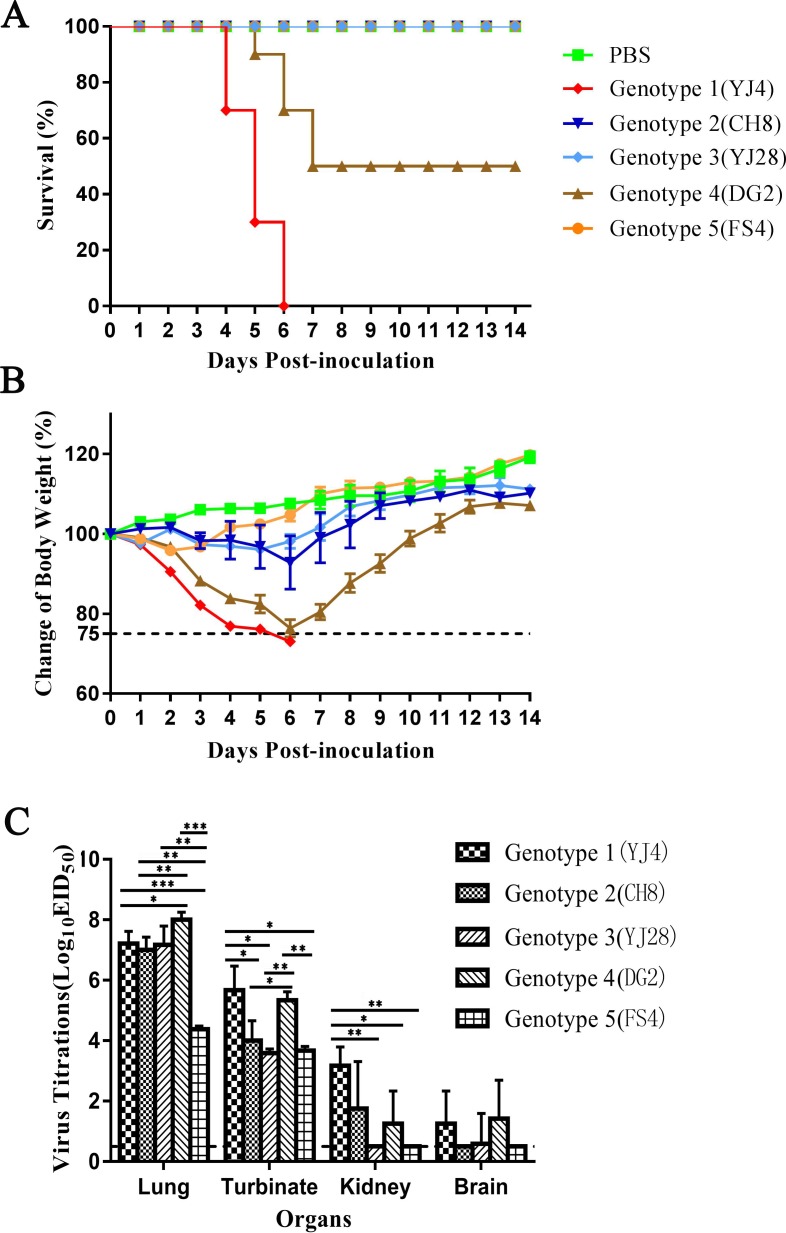
**Changes of body survival (A), weights (B), and virus titration (C) of mice.** 13 mice per group were anaesthetized and inoculated intranasally with 10^6.0^ EID_50_ viruses in 5 genotypes. The weight loss and survival of ten mice were recorded daily for a total of 14 days. Three mice were euthanized on day 3 post-inoculation, and the the lungs, kidneys, brains, and turbinates were collected, and the virus titrationd were tested in chicken embryos. Titers of viruses that are significantly different are marked as *, P<0.01; **, P<0.001; ***, P<0.0001. Statistical analysis was performed by one-way ANOVA.

Based on the results of phylogenetic analysis, we selected YJ4, CH8, YJ28, DG2 and FS4 from five genotypes to investigate the virulence, respectively. The results showed that mice infected with Genotype 3 (CH8) lost 21% of their body weight at most. YJ4 (Genotype 1) and DG2 (Genotype 4) could cause more than 25% weight loss and killed 100% and 50% of the mice, respectively (Fig 6). The results showed that the YJ4 (Genotype 1) and DG2 (Genotype 4) were considered to be the most pathogenic to mice. Organs from each mouse were collected to evaluate virus replication titration on day 3 post-inoculation. Virus was detected in all lungs and turbinates of the mice inoculated with 5 genotypic viruses. The titrations of the lungs inoculated with genotype 4 (DG2), 3 (YJ28), 1 (YJ4) and 2 (CH8) were significantly higher than genotype 5 (p<0.01), and the titrations of turbinates inoculated with genotype 4 and genotype 1 were signicantly higher than the remaining three genotypic groups. Furthermore, viruses were detected in the kidneys of three mice inoculated with YJ4, and in two mice inoculated with CH8 and DG2. The virus were also deteced in the brains of two mice inoculated with YJ4 and DG2, and one mice inoculated with YJ28. The mice inoculated with genotype 5 (FS4) present slight decrease of body weight, and the viruses can be detected only in the lungs and turbinates, not in the kidneys and brains.

## Discussion

Due to unique geographical and environmental factors, southern China is considered an important reservoir of influenza virus. In the first decade of the 21st century, Multiple lineages of IAVs-S have emerged and become established in pigs in southern China: classical swine H1N1 (CS), European avian-like H1N1 (EA) and triple-reassortant H1N2 viruses (TRIG). In 2001, the first case of infection with the EA-origin virus in pigs in Asia was reported in Hong Kong, and EA-origin viruses have since formed a stable phyletic clade in China [36]. In addition, TR-origin viruses have been regularly isolated from pigs in China since 2002 [37]. Since the pdm/09-origin virus outbreaks in humans, this virus has been repeatedly transmitted in pig herds [38–40]. Reassortant variants with pdm/09-origin gene segments and endemic genes were subsequently found in Asia [41–43]. The swine-origin H1N1 viruses were found reassorting with the H3N2 canine influenza viruses circulate endemically in Asian dogs [44]. Furthermore, the novel triple EA H1N1 and Human Like H3N2 reassortants, containing the CS H1N1 NS genes and the remaining five or four genes originating from H1N1/2009 pandemic, may have become established in pig herds in Southern China [40, 45]. Notably, the reassortant EA H1N1 viruses with EA-origin M gene, pdm/09-origin internal genes and CS-origin NS gene have been reported in human infections in Hunan, China [46].

In this study, phylogenetic analyses assigned the external genes of the novel isolates to the EA-origin H1N1 and/or human-origin H3N2, and the isolates were categorized into H1N1, H1N2 and H3N2. The regular isolation of H1N1 and H1N2 viruses demonstrates their continuing presence in pig herds, which means that the EA-origin variants reassorted with pdm/09-origin internal genes have become the major swine influenza lineage prevailing in southern China [41]. In addition, one H3N2 reassortant virus containing the pdm/09-origin internal genes was isolated in this study, indicating that the genotype of internal genes has reassorted with other endemic influenza viruses, indicating the pdm/09-origin internal genes affected the prevalence of H3N2 viruses in pig herds.

It has been reported that the pdm/09-origin internal genes has become predominant in pig herds in Guangdong [41]. All novel isolates possessed the pdm/09-origin PB2, PB1, PA and NP gene segments, indicating that the pdm/09-origin PB2, PB1, PA and NP genes had replaced the EA- or TR-origin gene segments. By contrast, the M genes were assigned to the pdm/09 lineage and EA lineage, partly due to the critical role of the pdm/09-origin M gene segment for high transmission efficiency in mammalian hosts[47]. The pdm/09-origin internal genes has already altered the IAV-S gene pool and influenced the prevalence of viruses in southern China. Moreover, all NS genes of the isolates belonged to the TR lineage and were assigned to the polyphyletic lineage formed in pigs in southern China, distinct from the TR-origin viruses found in the USA and Korea.

According to the phylogenetic analysis, the external genes of the isolates were assigned to the Eurasian avian-like swine (EA) H1N1 and/or human-like H3N2 lineages. On the other hand, the internal genes were assigned to pdm/09-like lineage(PB2, PB1, PA, NP, M), EA lineage(M) and/or TR lineage(NS). Five isolates in each genotype with the most representative functional mutations were selected for pathogenicity test in mice. The pathogenicity of genotype 1 and 2 were much higner than that of the other genotypes and could replicate systemically in mice. Especially, the genotype 1 virus could kill mice with 10^6.0^ EID_50_ viruses. Therefore, recombination of specific lineage of HA and NA genes could enhance the virulence in mice.

The molecular characteristics of amino acid residues at antigenic sites, host adaptation, virulence markers, and drug resistance were summarized. Amino acid residues at the receptor binding pocket of HA1 223Q (H3 sequence 226I) and 225G (H3 sequence 228S) were conserved in all isolates in this program and were predicted to have affinity for mammalian cell-surface receptors. Gly at position 222 in the HA protein has been reported in some patients with severe or fatal H1N1/2009 infection, and the D222G substitution in pdm/09-origin HA could alter receptor specificity and increase virulence in mice [48]. The E222G substitution was present in the HA genes of YJ4, YJ28 and DG2, but its significance in the EA-origin HA gene is unclear.

The functional sites in the pdm/09-origin internal genes were relatively conserved after 2009. Similar to pdm/09 in humans, 627E and 701D were present in the swine-origin pdm/09-origin PB2 gene segment, which is regarded as a determinant of host range and has been detected in the majority of avian viruses [49, 50]. These substitutions have been reported to have no major impact on virus replication or pathogenesis in mammalian models [51, 52]. By contrast, the T271A, T588I, G589R and Q590S substitutions were discovered in TR- and pdm/09-origin PB2, likely compensating for the lack of 627K and 701N in the pdm/09-origin PB2 protein [53]. Interestingly, N was found at position 701 in the FNPA PB2 gene and could contribute to the adaptation and pathogenesis of the virus to the mammalian hosts combined with other substitutions in this novel isolate.

The latest surveillance data showed that the pdm/09-origin variants emerged in central Mexico as a result of inter-hemispheric swine movements, which is closely related to long-distance pig transportation [54]. The mice experiment demonstrated that the novel reassortant viruses showed different pathogenicity, indicating a risk for the new potential pandemic. The alterations of IAV-S gene composition combined with the complex epidemic situation underlines the importance of continued swine surveillance in China to maintain public health.

This is the active surveillance to understand the evolution trend of IAV-S. However, there are two potential limitations that should be considered. First, the lack of detailed background information about the isolates could introduce interpretation bias into the results analysis. Second, the drug resistance, the receptor binding capacity, and antigenic characteristics of isolates have been predicted in the amino acid analysis, but the further experiments need be performed for verification.
